# Quarantine Experience-Based Differences in Factors Associated with Depression Among Koreans During the COVID-19 Pandemic

**DOI:** 10.3390/healthcare12212165

**Published:** 2024-10-30

**Authors:** Younghee Jeong, Moonkyoung Park

**Affiliations:** 1 Department of Nursing, Woosong College, Daejeon 34518, Republic of Korea; jeongyh@wsi.ac.kr; 2 College of Nursing, Chungnam National University, Daejeon 35015, Republic of Korea

**Keywords:** quarantine, depression, COVID-19, community health survey

## Abstract

Background/Objectives: The strict preventive measures implemented globally during the COVID-19 pandemic affected mental health, with most countries reporting a rise in depression and suicide. This study examined factors affecting depression among Korean adults based on quarantine experiences during 2020 and identified key areas for mental health support. Methods: Data were obtained from South Korea’s 2020 Community Health Survey. Responses from 219,228 adults (1893 with quarantine experience and 217,335 without) to questions about quarantine experience, depression (Korean version of PHQ-9 score ≥ 10 or a response of ‘several days’ or more on item 9), and individual/environmental factors were analyzed. Complex sampling analysis, including descriptive statistics and logistic regression, was conducted using SPSS 29.0. Results: This study found that 158 (weighted 7.5%) of those with quarantine experience, and 12,833 (weighted 5.8%) without quarantine experience, reported depression. Regardless of quarantine, being female, having a low income, a history of depression, and increased stress were associated with a higher depression rate, while good subjective health was linked to lower depression rates. Smoking, living-alone, and a lack of sleep among non-quarantined individuals were linked to increased depression risk, while adequate physical activity was linked to reduced risk. Interestingly, alcohol consumption and being overweight (BMI 23–<25) were associated with lower depression rates. Environmental factors, like unmet medical needs and reduced daily activity, were linked to increased depression. Strong social support and social distancing adherence were associated with reduced depression. Conclusions: These findings underscore the importance of targeted interventions considering quarantine experiences to reduce depression during pandemics.

## 1. Introduction

The novel infectious disease COVID-19, which emerged at the end of 2019, differed from previous epidemics in both its high transmissibility and mortality rates. From the onset of the outbreak, stringent public health measures were implemented globally [1,2]. While quarantine measures and stringent social distancing were crucial policies for preventing the spread of infection, they also brought about unexpected changes to individuals’ daily lives and negatively impacted mental health [3,4].

Anxiety about the infection and social distancing policies hardened the social atmosphere, and worsened public mental health [2,5]. Depression is experienced by approximately 5% of the global adult population [6]. Given its significant socioeconomic impact, including increased healthcare costs and suicide, early detection and treatment are essential [7,8]. In 2020, depressive symptoms or depression rates rose by 10–30% across most OECD countries compared to pre-pandemic levels, with a 36.8% increase in South Korea [9]. Depression also contributes to an increased risk of suicide. In 2020, South Korea’s suicide rate surged by 46% compared to previous years [10], indicating a severe decline in the mental health of its population. According to a systematic review by Wu et al., during the early stages of the COVID-19 pandemic, 44.8% of patients with chronic diseases, 41.7% of COVID-19 patients, 38.8% of individuals in quarantine, and 31.5% of the general public experienced depression [11]. Patients infected with COVID-19 experienced anxiety and depression due to the uncertainty of recovery, isolation, potentially transmitting the virus to others, and stigma. Furthermore, lingering symptoms such as fatigue and weakness post-recovery continue to contribute to psychological distress [12]. The general public experienced a deterioration in mental health due to the restrictions on daily life and prolonged indoor confinement imposed by COVID-19 containment policies [13,14,15,16,17,18]. The neologism “Corona Blues”, combining ‘COVID-19’ and ‘blues’, reflects a societal phenomenon highlighting the negative mental health issues experienced during the pandemic [19]. Psychological distress was more pronounced among women, younger individuals, and those of lower socioeconomic status. Factors such as marital status, social support, experiences related to COVID-19 infection, chronic illnesses, activity levels, and duration of isolation were also associated with the severity of mental health impacts [1,16,18].

However, most studies were conducted at the onset of the COVID-19 pandemic in early 2020 [20,21], and some research showed no correlation between containment policies and mental health outcomes [22]. These studies primarily focused on COVID-19 patients, individuals in quarantine, or the general public, limiting insights into the mental health of individuals’ post-quarantine. Therefore, this study aims to fill this gap by investigating the specific factors influencing depression among those who have been released from quarantine. Examining the relationship between quarantine experiences and mental health can reveal how such experiences impact long-term psychological well-being, thereby allowing for the identification of key factors for targeted support.

The socio-ecological model provides a theoretical framework integrating various theories on individual behavior and health. This explains how a combination of individual and external factors, including interpersonal, environmental, and societal determinants, can influence an individual’s health [23]. Depression can be influenced by various factors at the individual, interpersonal, organizational, and community levels, making it explicable within the socio-ecological model proposed by Bakhtari and colleagues [24].

Defining environmental factors as interpersonal, societal, and environmental influences, this study aims to examine the incidence of depression and the impact of personal and environmental factors on depression during the first year of the pandemic in 2020, when South Korea implemented its strictest social distancing measures. It used raw data from the 2020 Community Health Survey in South Korea (KCHS) [25] to identify the determinants of depression. These findings are intended to support public health policies during infectious disease pandemics like COVID-19, emphasizing the need for ongoing mental health management, particularly for persistent depression.

## 2. Materials and Methods

### 2.1. Research Design and Sample

This study was a secondary analysis conducted according to the guidelines for analyzing data from the 2020 KCHS [25]. The 2020 KCHS requested respondents to report their experiences of quarantine or hospitalization related to COVID-19 from January 2020 onwards. The data collection period was from 16 August to 31 October 2020, and interviewers visited selected households to conduct one-on-one interviews using laptops equipped with survey software. The study sample was selected through probability proportional-to-size sampling from households, with participants further chosen using systematic sampling methods. This research involved 229,269 adults aged 19 and above participating from 255 public health centers across all 17 provinces and cities in South Korea. The analysis focused on responses from 219,228 individuals regarding their COVID-19 quarantine experience, depressive symptoms, individual factors, and environmental factors. The data included 1893 individuals who had experienced quarantine or hospitalization and 217,335 individuals who had not been quarantined.

### 2.2. Measurements

The questions and response categories used to assess individual and environmental factors are detailed in Table 1. The experience of self-quarantine or hospitalization was evaluated using a dichotomous response (yes/no) to the question “Have you ever been quarantined or hospitalized due to COVID-19?” Depression was assessed using the Patient Health Questionnaire-9 (PHQ-9), which was standardized in Korean by Choi and colleagues for screening major depressive disorder in the Korean population. At the time of standardization, the reliability of the PHQ-9 was reported to be 0.85 [26]. In this study, depression was defined as a score of 10 or higher on the PHQ-9, or a response of “several days” to “nearly every day” on item 9 of the questionnaire.

### 2.3. Ethical Considerations

The 2020 KCHS is a government-approved statistical investigation conducted by the Korea Disease Control and Prevention Agency under the Public Health Act. It operates on a four-year survey cycle, with the 2020 survey being part of the third cycle of rotational studies. The raw data, made public in accordance with the relevant procedures and regulations, were accessed and utilized after completing the necessary steps. Employing secondary data from the 2020 KCHS, this study was exempted from review by the Institutional Review Board (No. 202406-SB-076-01).

### 2.4. Statistical Analysis

In this study, complex sample statistics were analyzed using SPSS software (version 29.0; IBM, Armonk, NY, USA) in accordance with the guidelines provided by the KCHS. Descriptive statistics, including frequencies and weighted percentages, were used to examine individual and environmental factors across groups based on whether they had experienced quarantine. Logistic regression analysis was conducted to identify differences in individual and environmental factors associated with depressive symptoms related to quarantine experiences.

## 3. Results

### 3.1. Descriptive Analysis of Quarantine Experience and Individual and Environmental Factors

Among the 1893 adults who experienced quarantine, 158 individuals (7.5%) reported depression. In contrast, 12,833 individuals among those who did not experience quarantine (5.8% of 217,335) exhibited depression. The distribution of individual and environmental factors associated with depression, differentiated by quarantine experience, is shown in Table 2.

### 3.2. Associated Factors Between Individual and Environmental Factors According to Quarantine Experience

Several factors were associated with depression in the quarantine group. Individual factors such as high subjective stress, previous experience of depression, low household income, and being female increased the likelihood of depression. Conversely, moderate-to-good subjective health was associated with a lower likelihood of depression. However, no significant environmental factors influencing depression were identified.

In the no-quarantine group, individual factors such as previous experiences of depression, high subjective stress, low income, current smoking, being a young adult aged 19–29 years, having an education level of middle school or below, living alone, appropriated sleep hours, and being female were associated with an increased likelihood of depression. Conversely, having moderate to good subjective health, being aged 50–64 years, engaging in adequate physical activity, being overweight, and alcohol consumption were associated with a decreased likelihood of depression. Among the environmental factors, experiencing unmet medical care and a significant decrease in changes in daily life compared to pre-pandemic times were associated with an increased likelihood of depression, whereas compliance with social distancing and having someone help when needed were associated with a decreased likelihood of depression (Table 3).

## 4. Discussion

This study analyzed individual and environmental factors related to the incidence of depression among adults during the 2020 COVID-19 pandemic, using data from the 2020 Community Health Survey. The findings showed that the prevalence of depression was 7.5% in the quarantine-experience group and 5.8% in the non-quarantine group, indicating that the factors influencing depression varied depending on quarantine experience.

The incidence of depression in this study was slightly lower than that reported in China (8.1% in the self-quarantine group, 38.2% in the hospital quarantine group, and 7.5% in the general population) [28], which differs from the findings of Wu et al.’s meta-analysis (41.7% in COVID-19 patients, 38.8% in quarantined individuals, and 31.0% in the general population) [11]. The incidence of depression in the early stages of the COVID-19 pandemic was reported to have increased by approximately 26–40% compared to pre-pandemic levels [11,20,29]. However, as national prevention policies were gradually established and infection rates declined, a corresponding decrease in the incidence of depression was observed [30]. Countries that implemented stringent prevention policies from the early stages of the pandemic observed relatively lower depression rates compared to those without such policies [31]. South Korea was one of the countries applying strong and systematic prevention policies from the early stages of the pandemic. Moreover, the country swiftly and flexibly adjusted its prevention measures according to the characteristics of the outbreak [32]. Resultantly, the incidence of depression was relatively lower. Meanwhile, as social distancing measures increased the time spent at home, they led to certain positive outcomes, such as maintaining a better work–life balance, enhancing closeness with family and friends, and experiencing greater temporal flexibility. These factors have been reported to elicit some positive responses [33,34]. While the negative impact of the pandemic on mental health is evident, utilizing these buffering factors and incorporating them into response management during the early stages of a pandemic could contribute to maintaining and enhancing mental health.

However, in this study, individuals who experienced quarantine related to COVID-19 had a higher incidence of depression than the general population, which is consistent with findings from previous research [11,28]. Quarantine itself can have negative psychological effects, and when combined with factors such as fear of infection, duration of quarantine, loneliness, inaccurate information or stigma, and economic losses [1], it can further exacerbate stress, and potentially increase depression. Although the participants in this study were released from quarantine, they still exhibited a higher incidence of depression than the general population. This could be associated with factors such as uncertainty of the disease course, traumatic memories related to the infection, loneliness due to physical isolation, stigma, neuropsychiatric syndromes caused by immune responses to the infection, or brain damage induced during the treatment process [3]. For individuals requiring quarantine, it is therefore important to pay close attention not only to their mental health during the quarantine period, but also to any long-term changes in their mental health after the quarantine has been lifted.

According to the study results, regardless of quarantine experience, factors such as being female, having a low income, a history of depression, and high levels of subjective stress were associated with a higher incidence of depression. Conversely, a positive perception of one’s health was associated with a reduced incidence of depression. Given that women are more vulnerable to depression than men due to society-driven risk factors such as female hormones, physical strength, and personality traits [35], it is not surprising that several studies have reported women being more vulnerable to depression during the COVID-19 pandemic. Factors such as concerns about infection during pregnancy and childbirth, limited access to healthcare, the burden of household chores and family care, restricted time for education or career development, and exposure to partner violence have been identified as contributing to the deterioration of women’s mental health [20,34,36,37,38]. Low income levels were also a factor increasing the incidence of depression, while financial difficulties are associated with deteriorating mental health [39]. Even after controlling for pre-COVID-19 income levels, concerns or burdens related to finances during the pandemic can be considered sensitive indicators related to an increase in depression [40]. Most public health policies that restrict daily activities also limit economic activities, leading to economic stress, which, in turn, contributes to the incidence of depression. Vulnerable populations such as women and those with a low socioeconomic status are more likely to experience social inequalities, which can negatively impact their mental health [41]. To maintain mental health, it is thus essential to implement comprehensive measures that include universal access, alongside tailored social, medical, and economic support, considering the specific needs of vulnerable populations. Moreover, these interventions should be detailed and multileveled to effectively address the diverse challenges faced by these groups.

Individuals who experienced depression before the pandemic were at higher risk of developing depression during the pandemic [42]. In this study, individuals with a history of depression had a five- to eight-times higher risk of developing depression. This suggests that restricted social interactions and limited activities during the pandemic may have heightened feelings of isolation and loneliness, potentially worsening pre-existing mental health conditions [20,42]. In addition, a high level of subjective stress is a factor that increases the incidence of depression. People tend to be more vulnerable to depression when they have a heightened sensitivity to stress and reduced resilience [43]. During the COVID-19 pandemic, individuals who experienced quarantine were psychologically affected by stressors such as anxiety about the infection, restrictions on social activities, lack of information, economic difficulties, and stigma [1,16]. From a neurocognitive perspective, abnormal cognitive responses to stress can lead to chronic autonomic nervous system dysfunction, dysregulation of inflammatory processes, and changes in the neuroendocrine system, all of which can alter the brain’s neural circuitry. This can increase maladaptive stress reactivity and the risk of depression [44]. Therefore, cognitive interpretations of stressors and the resulting physiological responses may be key factors in the development of depression. As such, identifying the sources of stress and implementing psychological interventions for individuals who may be psychologically vulnerable should be done from the early stages of a pandemic.

Subjective health perceptions mediate the relationship between physical and mental health [45]. In other words, how individuals perceive their own health is closely related to their mental health [46]. A positive perception of one’s own health can enhance mental well-being, and reduce symptoms of depression [47]. The findings of this study also suggest that a positive perception of health status can serve as a protective factor against the onset of depression.

Among individuals without quarantine experience, certain personal factors such as younger age, living alone, low educational attainment, current smoking, and poor sleep habits were associated with an increased risk of depression. In contrast, middle age (50–64 years), alcohol consumption, adequate physical activity, and being overweight were factors that decreased the risk of depression. Among the environmental factors, unmet medical needs and significant restrictions on daily life were associated with an increased risk of depression, while having someone to seek help from and strict adherence to social distancing policies were factors reducing this risk.

During the COVID-19 pandemic, several other countries also reported that younger age groups, lower educational levels, and individuals living alone were more vulnerable to depression [20,36], aligning with our findings. This highlights the need for careful consideration of sociodemographic factors when planning psychological interventions for depression in the general population.

The lifestyle changes during the COVID-19 pandemic led to negative shifts in health behavior. In this study, the smoking rate among the general population during the COVID-19 pandemic was 37.7%, similar to pre-pandemic levels. Typically, smokers tend to exhibit higher levels of depression than non-smokers [48]. Given that smoking can be used as a way to regulate emotions [49], this indicates a deeper connection between smoking and mental health issues such as depression; efforts to reduce smoking are thus necessary as part of depression management.

Sleep duration is recognized as a modifiable factor associated with depression [50]. One meta-analysis during the COVID-19 pandemic found that 35.7% of all participants and 32.3% of the general population experienced sleep problems [51]. Changes in sleep duration, such as increases or decreases, are closely associated with mental health issues, including depression, anxiety, and loneliness [52]. In this study, although the number and causes of change in sleep duration compared with the pre-pandemic period were not identified, 38% of the general population reported inadequate sleep duration, which was a factor that increased depression. Therefore, efforts to improve sleep duration and quality may contribute to enhancing mental health.

This study found that smoking was associated with an increase in depression, whereas alcohol consumption was associated with a decrease in depression. A cohort study using a marginal structural model approach demonstrated that not engaging in heavy episodic drinking (defined as drinking less than once per week on average) could reduce the risk of depression [53]. According to the 2019–2020 U.S. National Alcohol Surveys, during the early stages of the COVID-19 pandemic, as depression and anxiety increased, there was an increase in drinking behavior aimed at alleviating worries, which was associated with depression [54]. According to the ‘Self-Medication Hypothesis’, individuals may drink alcohol as a form of self-prescription to cope with negative emotions. The use of alcohol to escape or cope with external stressors can serve as a negative reinforcer, perpetuating alcohol use, and potentially exacerbating depression and anxiety [55]. In stressful situations like the pandemic, drinking to alleviate symptoms of depression and anxiety may lead to alcohol misuse or dependence [1], thereby further increasing depression or anxiety. Although moderate alcohol consumption may reduce the risk of depression, stronger associations between alcohol use and increased risk of depression have been reported [56]. Therefore, further research using various methodological approaches is required to comprehensively explore this relationship.

Another factor associated with reduced depression in this study was being overweight (based on the Asian BMI standard of 23–24.9). The relationship between body weight and depression varies significantly depending on cultural contexts. In Western societies, a thin body ideal is often promoted and being overweight or obese can lead to stigma, increasing the risk of body dissatisfaction and depression [57]. In contrast, in some African and Asian cultures, a larger body size is associated with health and wealth, which may weaken or eliminate the link between obesity and depression [58]. During the COVID-19 pandemic in Vietnam, depression was reported to increase among underweight elderly individuals, likely due to the perception that being thin is associated with poor health [59]. Traditionally, being overweight has been considered ideal and healthy in Korea, and there is a tendency for both elderly women [60] and men [61] to underestimate their actual weight status. While obesity or severe obesity increases the risk of ICU admission and mortality in the event of COVID-19 infection, being overweight does not present the same level of risk [62]. Among elderly patients with COVID-19 in Korea, the risk of severe infection and mortality tended to be lower in those who were overweight [63]. According to a representative study in Germany, depression increased and health-related quality of life was perceived as lower only in cases of obesity, compared to normal weight or overweight individuals [64]. Based on these findings, it can be speculated that overweight individuals in Korea may have perceived themselves as healthy, which may contribute to the reduction in the incidence of depression. As such, perceptions of one’s own health in a pandemic situation need to be further explored within different cultural contexts.

The present study found adequate physical activity to have a significant impact on reducing the incidence of depression. Several meta-analyses have demonstrated that physical activity is effective in preventing depression [65] and improving depressive symptoms [66]. Furthermore, the combination of medication therapy and exercise has been suggested to be even more effective in alleviating depression [67]. However, the social distancing policies implemented during COVID-19 led to restrictions on physical activity, and reduced levels of physical activity were associated with an increase in mood disorders such as depression and anxiety [68]. Maintaining adequate physical activity even during a pandemic could thus help reduce depression [69]. In other words, when daily life is restricted due to a pandemic, it is essential to develop strategies allowing individuals to safely maintain physical activity in their daily routines (e.g., web-based virtual exercise programs).

Unmet medical care increased the incidence of depression in the group without quarantine experience. When individuals need medical services but their expectations for accessing them are not met, they may experience disappointment and stress, which can contribute to the development of depression [70]. In Korea, quarantined individuals exposed to COVID-19 received active support during quarantine. This included health monitoring by healthcare professionals or public health authorities and guidance on accessing medical services when needed, along with other relevant COVID-19 information [71]. However, among the general population, there was a tendency to postpone or forgo scheduled medical appointments due to fear and concern about COVID-19 infection [72]. This tendency was more pronounced among the elderly, individuals with chronic conditions, low-income groups, and those with lower education levels, making them more likely to experience unmet medical needs [73]. To address these unmet needs, it is necessary to establish measures that consider the specific demands and characteristics of these populations, such as telemedicine and remote health information consultations.

Individuals who perceived significant restrictions in their daily lives compared to the pre-pandemic period were found to experience an increase in depression. During the COVID-19 pandemic, social distancing policies led to a marked reduction in regular indoor and outdoor activities, as well as interpersonal interactions, which increased feelings of frustration, isolation, and loneliness. These negative emotions may contribute to an increase in depression [13,42]. Online psychosocial interventions [74] and social network services can effectively mitigate feelings of isolation and loneliness [1]. During the early stages of the pandemic, the South Korean government actively used social media platforms, virtual spaces, and the creation of psychosocial support content to promote mental health. The government also leveraged partnerships with hospitals, universities, and public–private institutions. Mental health professionals provided various forms of support, including online platforms and telephone counseling, which proved effective in reducing depression [4]. Active policies aimed at providing psychosocial support during social distancing are essential to reduce depression. However, as mental health status can vary depending on the duration of the pandemic, it is also necessary to have flexible psychosocial support plans that can adapt to changing circumstances.

When sudden quarantine situations related to COVID-19 exposure arose, having someone to seek help from was associated with a reduced incidence of depression. Feeling meaningfully connected to someone can significantly contribute to the promotion of mental health [75]. In unexpected crisis situations like COVID-19, social support can serve as a protective factor against loneliness [76] and reduce the likelihood of depression [77]. Therefore, social support can be considered a predictive factor for depression [75]. However, while social support provided remotely during the pandemic was effective in some cases [1,74], there were also instances where it was not as effective [78]. Therefore, further research is required to evaluate the effectiveness of remote social support during pandemic situations.

Voluntary compliance with social distancing is crucial in controlling a pandemic. In the present study, proper adherence to social distancing measures was associated with a reduced incidence of depression. Compliance with social distancing is closely related to perceptions of COVID-19. Insufficient awareness of COVID-19 can increase anxiety levels [79], which may increase the risk of depression. Providing accurate and reliable information, along with fostering a sense of civic duty, are key factors in promoting adherence to social distancing guidelines [80]. Therefore, the government has an obligation to promptly correct misinformation related to COVID-19 and provide accurate and practical guidelines [79].

This study has several limitations. Patients with chronic diseases may experience increased worry and anxiety about infection due to the higher mortality risk associated with COVID-19, which could lead to a higher incidence of depression [81]. However, this study did not find evidence that depression was more prevalent among individuals with chronic illnesses; it may therefore be necessary to investigate depression-related factors based on health status. In addition, the data used in this study were from the first year of the COVID-19 pandemic, August to October 2020, which limited the ability to track changes in factors affecting depression throughout the entire pandemic period. Moreover, the original data did not distinguish between infected participants and those who only experienced quarantine, and no data from period after quarantine were provided, limiting the interpretation of the results. The generalizability of the study findings may also be affected by the potential for recall bias due to self-reported data, the inability to establish causality due to the cross-sectional design, non-responses, and social desirability bias, as well as regional differences. This study also did not fully incorporate factors based on an ecological model. Therefore, future research should address these limitations by employing longitudinal study designs, collecting data across different stages of the pandemic, distinguishing between infection and quarantine experiences, and integrating a comprehensive ecological approach to better understand the complex factors influencing mental health during and after pandemics. Despite its limitations, this study is significant because it utilized nationally representative data to provide evidence for developing interventions for depression management based on quarantine experiences.

## 5. Conclusions

This study identified factors associated with depression among Korean adults during the initial phase of the COVID-19 pandemic, focusing on those who experienced quarantine versus those who did not. The findings revealed that high subjective stress, history of depression, low income, and female gender were significant predictors of increased depression, regardless of quarantine experience. For individuals who were not quarantined, additional factors such as smoking, inadequate sleep duration, restrictions on daily activities, and unmet medical needs contributed to higher depression rates. Conversely, alcohol consumption, adequate physical activity, being overweight, social support, and adherence to social distancing measures were associated with lower depression rates. In conclusion, when implementing targeted interventions or policies to address depression during sudden pandemics such as COVID-19, it is crucial to consider not only the sociodemographic characteristics of individuals, but also their quarantine status or experience to ensure effective and differentiated responses.

## Figures and Tables

**Table 1:** Measurements of individual and environmental factors.

Variables	Measurement
Individual factors	
Gender	Male, female
Age	The age groups, reclassified based on productive activities according to ‘The National Atlas of Korea III’ [27], are as follows: 19–29 years (young adulthood), 30–49 years (adulthood), 50–64 years (late adulthood) and 65 years and older.
Household type	The single question to assess the type of household residence has the following available responses: ‘Living alone’ or ‘Not living alone’.
Household income	Classified as ‘less than 2 million won’, ‘2 million to less than 4 million won’, and ‘4 million won or more’.
Education level	Classified as either ‘Below middle school graduation’ or ‘High school graduation or higher’.
Current smoking	The single question “Have you smoked in the past 30 days?” has responses classified as ‘Yes’ for those who smoke daily or occasionally and ‘No’ for those who do not smoke.
Current alcohol consumption	The single question ‘Have you consumed alcohol in the past 30 days?’ classifies responses as ‘Yes’ for those who have consumed alcohol once a month or more and ‘No’ for those who do not drink.
Diabetes	Responses are classified as ‘Yes’ if diagnosed with diabetes or taking diabetes medication or insulin and ‘No’ if not.
Hypertension	Responses are classified as ‘Yes’ if diagnosed with hypertension or taking anti-hypertensive medication and ‘No’ if not.
Body mass index	Responses are classified into normal (<23 kg/m^2^), overweight (≥23 kg/m^2^ and <25 kg/m^2^), or obese (≥25 kg/m^2^).
Physical activity	Responses are classified as ‘Adequate’ if engaging in at least 150 min of moderate physical activity or at least 75 min of vigorous physical activity per week, and ‘Not adequate’ otherwise.
Subjective health status	The single question assesses subjective health status. Available responses are classified as ‘good’ for excellent or good, ‘moderate’ for moderate, and ‘poor’ for poor or very poor.
Subjective stress status	The single question assesses subjective stress status. Available responses are classified as ‘Low’ for feeling little stress and ‘High’ for feeling very stressed.
Depressed experience	Responses to the question “Have you felt sad or hopeless for more than two weeks in the past year?” are classified as ‘Yes’ or ‘No’.
Sleep	Responses to the question regarding average sleep duration over the past week are classified into ‘less than 7 h’, ‘7 to 9 h’, and ‘more than 9 h’.
**Environmental factors**	
Unmet medical needs	Responses to the single question, “Have you needed but not received medical treatment (testing or therapy) in the past year?” are classified as ‘Yes’ if the answer is ‘yes’, and ‘No’ if the answer is ‘no’ or ‘it was not necessary’.
Changes in daily life	The change in daily activity level was measured on a scale of 0 to 100 in response to the question ‘How does your current daily activity level compare to before the COVID-19 pandemic?’ A score of 100 means that daily life is the same as before the pandemic, and a score of 0 means that daily life has completely stopped. In this study, when comparing the daily life with before the pandemic, it was classified as ‘0 to 30 points: significantly limited’, ‘40 to 60 points: slightly limited’, and ‘70 to 100 points: similar’.
Compliance with social distancing	Compliance with social distancing is categorized as ‘Well’ for responses ‘Yes’ and ‘Very much so’, and as ‘Not complying’ for the response ‘No’.
Social support	Responses to the question, “How many people can you urgently ask for help if you are placed in quarantine treatment or self-isolation?” are classified as ‘Yes’ if the answer is one or more, and ‘No’ if the answer is none.
**Depression**	Responses classified as ‘Yes’ for the presence of depression if the PHQ-9 score is 10 or above, or if the response to item 9, “thoughts that you would be better off dead or hurting yourself in some way” ranges from “several days” to “nearly every day”. Classified as ‘No’ for the absence of depression if the score is below 10.

**Table 2:** Characteristics by quarantine experience.

Variables	Categories	Quarantine Experience (n = 1893)	No Quarantine Experience (n = 217,335)
		N * (Weighted %)	N * (Weighted %)
Individual factors			
Gender	Male	831 (47.5)	100,332 (50.0)
	Female	1062 (52.5)	117,003 (50.0)
Age (in years)	19~29	322 (24.1)	25,168 (17.0)
	30~49	577 (39.7)	59,311 (35.4)
	50~64	538 (23.7)	66,747 (28.3)
	≥65	456 (12.5)	66,109 (19.3)
Household type	Living alone	175 (8.6)	32,632 (12.1)
	Not-living alone	1718 (91.4)	184,703 (87.9)
Household income (10,000 won)	<200	478 (16.9)	67,125 (20.7)
	≥200~<400	528 (26.4)	64,748 (29.8)
	≥400	887 (56.7)	85,462 (49.5)
Education level	≤Middle school	826 (35.1)	69,356 (19.1)
	≥High school	1067 (64.9)	147,979 (80.9)
Current smoking	Yes	682 (37.5)	77,427 (37.7)
	No	1211 (62.5)	139,908 (62.3)
Alcohol consumption	Yes	745 (30.7)	89,885 (33.7)
	No	1148 (69.3)	127,450 (66.3)
Diabetes	Yes	198 (7.3)	25,112 (9.0)
	No	1695 (92.7)	192,223 (91.0)
Hypertension	Yes	411 (13.7)	59,294 (20.9)
	No	1482 (86.3)	158,041 (79.1)
Body mass index (kg/m^2^)	<23	833 (45.2)	97,479 (45.0)
	≥23~<25	448 (22.0)	53,490 (24.0)
	≥25	612 (32.8)	66,366 (31.0)
Physical activity	Adequate	635 (33.2)	63,200 (29.1)
	Not adequate	1258 (66.8)	154,135 (70.9)
Subjective health status	Good	904 (52.9)	105,591 (53.0)
	Moderate	687 (36.2)	84,161 (37.8)
	Poor	302 (10.9)	27,583 (9.2)
Subjective stress status	High	556 (32.2)	48,109 (25.5)
	Low	1337 (67.8)	169,226 (74.5)
Depressed experience	Yes	186 (9.6)	11,923 (5.8)
	No	1707 (90.4)	205,412 (94.2)
Sleep (in hours)	<7	724 (35.4)	80,888 (35.1)
	7~9	1096 (60.8)	130,408 (62.0)
	>9	73 (3.8)	6039 (2.9)
**Environmental factors**			
Unmet medical needs	Yes	657 (27.6)	9734 (4.3)
	No	1236 (72.4)	207,601 (95.7)
Social support	Yes	1566 (86.2)	179,731 (84.3)
	No	327 (13.8)	37,604 (15.7)
Changes in daily life	Significantly limited	1136 (53.4)	44,425 (22.4)
	Slightly limited	484 (30.8)	98,862 (46.6)
	Similar	273 (15.7)	74,048 (30.9)
Compliance with social distancing	Well	1627 (84.5)	194624 (88.64)
	No	266 (15.5)	22,711 (11.4)
**Depression**	Yes	158 (7.5)	12,833 (5.8)
	No	1735 (92.5)	204,502 (94.2)

Note 1. * = Unweighted.

**Table 3:** Factors related to depression according to quarantine experience.

Variables	Categories	Quarantine Experience (n = 1893)		No Quarantine Experience (n = 217,335)	
		OR	95% CI	OR	95% CI
Individual factors					
Gender (Ref. = Male)	Female	2.17	1.04–4.51	1.62	1.50–1.76
Age (year) (Ref. ≥ 65)	19~29	1.46	0.52–4.10	1.34	1.19–1.50
	30~49	0.93	0.34–2.51	0.98	0.88–1.08
	50~64	0.95	0.41–2.17	0.87	0.80–0.94
Household type (Ref. = Not-living alone)	Living alone	1.00	0.49–2.07	1.27	1.18–1.36
Household income (10,000 won) (Ref. ≥ 400)	<200	2.26	1.09–4.66	1.80	1.66–1.95
	≥200~<400	1.62	0.86–3.05	1.33	1.23–1.43
Education level (Ref. ≥ High school)	≤Middle school	0.79	0.40–1.54	1.33	1.23–1.44
Current smoking (Ref. = No)	Yes	1.09	0.51–2.30	1.48	1.36–1.60
Current alcohol consumption (Ref. = No)	Yes	1.19	0.71–2.00	0.93	0.87–0.99
Diabetes (Ref. = No)	Yes	1.02	0.45–2.34	1.08	0.99–1.17
Hypertension (Ref. = No)	Yes	1.65	0.77–3.53	1.02	0.95–1.09
Body mass index (kg/m^2^) (Ref. < 23)	≥23~<25	1.13	0.60–2.16	0.87	0.81–0.93
	≥25	1.08	0.60–1.96	0.96	0.90–1.02
Physical activity (Ref. = Not adequate)	Adequate	1.16	0.70–1.92	0.94	0.88–0.99
Subjective health status (Ref. = Poor)	Good	0.20	0.09–0.41	0.25	0.23–0.27
	Moderate	0.46	0.24–0.88	0.42	0.39–0.45
Subjective stress status (Ref. = Low)	High	3.82	2.23–6.53	4.12	3.90–4.36
Depressed experience (Ref. = No)	Yes	5.39	3.15–9.21	8.06	7.56–8.60
Sleep (in hours) (Ref. > 9)	<7	0.94	0.53–1.65	1.21	1.15–1.28
	7~9	1.65	0.62–4.41	1.64	1.44–1.87
**Environmental factors**					
Unmet medical needs (Ref. = No)	Yes	1.02	0.52–1.97	1.91	1.93–2.10
Social support (Ref. = No)	Yes	0.91	0.47–1.77	0.79	0.74–0.84
Changes in daily activity level (Ref. = Similar)	Significantly decreased	2.08	0.95–4.54	1.15	1.07–1.24
	Slightly decreased	0.70	0.29–1.68	0.96	0.90–1.03
Compliance with social distancing (Ref. = No)	Well	0.97	0.53–1.80	0.70	0.65–0.75

Note 2. CI = confidence interval; OR = odds ratio; Ref. = reference.

## Data Availability

Publicly available datasets were analyzed in this study. These data are available at https://chs.kdca.go.kr/chs/main.do, accessed on 1 September 2024.
